# Homogeneous and heterogeneous risk and prognostic factors for lung metastasis in colorectal cancer patients

**DOI:** 10.1186/s12876-022-02270-5

**Published:** 2022-04-18

**Authors:** Hongmei Wang, Xuefeng Shan, Min Zhang, Kun Qian, Zhengze Shen, Weiying Zhou

**Affiliations:** 1 Department of Pharmacology, College of Pharmacy, Chongqing Medical University, 1 Yixueyuan Road, Yuzhong District, Chongqing, 400016 China; 2 Chongqing Key Laboratory of Drug Metabolism, Chongqing Medical University, Chongqing, 400016 China; 3 Key Laboratory for Biochemistry and Molecular Pharmacology of Chongqing, Chongqing Medical University, Chongqing, 400016 China; 4 Department of Pharmacy, The First Affiliated Hospital of Chongqing Medical University, Chongqing, 400016 China; 5 Department of Epidemiology and Health Statistics, School of Public Health and Management, Chongqing Medical University, Chongqing, 400016 China; 6 Department of Gastrointestinal Surgery, The First Affiliated Hospital of Chongqing Medical University, Chongqing, 400016 China; 7 Department of Pharmacy, Yongchuan Hospital of Chongqing Medical University, 439 Xuanhua Road, Yongchuan District, Chongqing, 402160 China

**Keywords:** Colorectal cancer, Lung metastasis, Risk factors, Prognosis, SEER

## Abstract

**Background:**

The lung is one of the most frequent distant metastasis sites in colorectal cancer (CRC) patients; however, lung metastasis risk and prognostic factors have not been comprehensively elucidated. This study aimed to identify the homogeneous and heterogeneous lung metastasis risk and prognostic factors in CRC patients using the Surveillance, Epidemiology, and End Results (SEER) database.

**Methods:**

CRC patients registered in the SEER database between 2010 and 2016 were included to analyse risk factors for developing lung metastasis by using univariable and multivariable logistic regression. Patients diagnosed between 2010 and 2015 were selected to investigate prognostic factors for lung metastasis by conducting Cox regression. Kaplan–Meier analysis was used to estimate overall survival outcomes.

**Results:**

A total of 10,598 (5.2%) patients with synchronous lung metastasis were diagnosed among 203,138 patients with CRC. The median survival time of patients with lung metastasis was 10.0 months (95% CI 9.6–10.5 months). Older age, unmarried status, uninsured status, poor histological differentiation, more lymphatic metastasis, CEA positivity, liver metastasis, bone metastasis and brain metastasis were lung metastasis risk and prognostic factors. Black patients and those with left colon, rectum, and stage T4 disease were more likely to develop lung metastasis, while patients with right colon cancer and no surgical treatment of the primary tumour had poor survival outcomes.

**Conclusion:**

The incidence of lung metastasis in CRC patients was 5.2%. CRC patients with lung metastasis exhibited homogeneous and heterogeneous risk and prognostic factors. These results are helpful for clinical evaluation and individual treatment decision making.

**Supplementary Information:**

The online version contains supplementary material available at 10.1186/s12876-022-02270-5.

## Introduction

Colorectal cancer (CRC) has become the third most commonly diagnosed cancer worldwide and is the second leading cause of cancer-related death [1]. Patients with localized stage CRC commonly have a 90% 5-year survival rate; however, the survival rate worsens when the cancer spreads to distant organs [2, 3]. The lung is one of the most common distant metastasis sites in CRC patients. It has been reported to be the second most common metastatic site [3, 4]. Previous studies revealed that the incidence of lung metastasis in CRC patients ranges from 2.40 to 11.0% [5, 6]. Early detection of the high-risk population susceptible to lung metastasis is important for clinical decision-making. Chest CT, 18F-FDG-PET/CT, puncture biopsy through the wall of the chest, and bronchoscopy are commonly applied for the detection of lung metastasis [7, 8]. However, these examinations commonly involve exposure to radioactivity and are invasive and expensive, increasing the economic burden on patients. Therefore, it is necessary to identify risk factors to improve lung metastasis screening in CRC patients.

CRC patients with lung metastasis usually have poor survival outcomes. A previous study revealed that the 1-year cause-specific survival rate of CRC patients with and without lung metastasis was 55.5% and 90.2%, respectively, which was worse than that of patients with liver metastasis [3]. CRC patients with different pathological or clinical characteristics usually show different prognoses. Although some studies have investigated the risk factors for distant metastases (such as liver metastasis) in CRC, the lung metastasis risk and prognostic factors are still unclear [6, 9], and homogeneous and heterogeneous lung metastasis risk and prognostic factors have not been explored. Identifying these specific factors associated with lung metastasis will help clinicians identify high-risk patients.

The purpose of this study was to analyse the risk factors for lung metastasis and estimate the lung metastasis-associated prognosis in patients newly diagnosed with CRC based on data from the Surveillance, Epidemiology, and End Results (SEER) database. We further identified the heterogeneous and homogeneous risk and prognostic factors.

## Methods

### Population

**Fig. 1:**
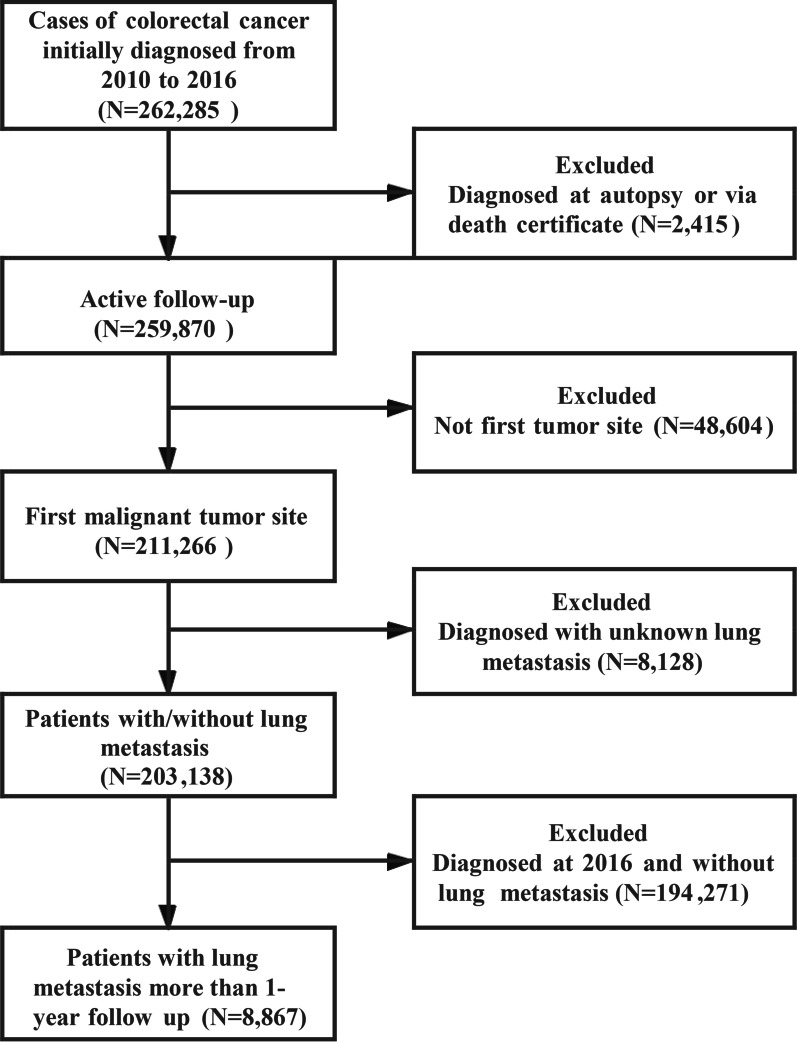
Flowchart of colorectal cancer patient selection

In this population-based study, CRC patient data were acquired from a US National Cancer Institute (NCI) open public database, the SEER database. SEER*Stat version 8.3.5 (https://seer.cancer.gov/seerstat/) (Information Management Service, Inc. Calverton, MD, USA) was used to generate the patient list. CRC patients diagnosed with lung metastasis between 2010 and 2016 were included in this study. Patients who were diagnosed at autopsy or via a death certificate were excluded. Patients with unspecified follow-up, a primary tumour outside of the colorectal region, and unavailable lung metastasis information were excluded. A flowchart showing the patient inclusion and exclusion process is presented in Fig. 1. Patients newly diagnosed with CRC between 2010 and 2016 were used to analyse lung metastasis risk factors, and patients diagnosed from 2010 to 2015 with a follow-up of at least 1 year were used to investigate the overall survival rate after lung metastasis.

### Statistical analysis

This study included the following variables: age (< 50, 51–60, 61–70, 71–80, 81–90, ≥ 91); sex (male and female); race (white, black, other (American Indian/Alaska Native and Asian or Pacific Islander); marital status (unmarried and married); insurance status (uninsured and insured); site of primary tumour (left colon, right colon and rectum); histological types (Grade I, Grade II, Grade III, Grade IV); N stage (N0, N1, N2); T stage (T1, T2, T3, T4); carcinoembryonic antigen (CEA) (negative, positive); without or with liver metastasis; without or with bone metastasis; without or with brain metastasis; and surgical treatment for the primary cancer (yes or no).

Quantitative data are presented as the mean ± standard deviation (SD), and categorical data are described as numbers and percentages (N, %). Univariate and multivariate logistic regression were used to identify the factors associated with synchronous lung metastasis. The Kaplan–Meier method was used to estimate overall survival outcomes. Univariate and multivariate Cox regression analyses were conducted to identify potentially associated prognostic factors. Statistically significant levels were two-tailed and set at *P* < 0.05. Statistical analyses were conducted with the IBM Statistical Package for the Social Sciences (SPSS) version 23.0 software package for Windows (SPSS, Inc., Chicago, IL, USA). MedCalc 18.0 was used to generate survival curves.

## Results

### Patient characteristics

A total of 203,138 CRC patients were initially identified between 2010 and 2016. Of these patients, 10,598 (5.2%) patients were initially diagnosed with lung metastasis, and 192,540 (94.8%) patients were without lung metastasis. The mean age of all patients was 64.88 ± 14.32 years. A total of 105,727 (52.0%) patients were male. A total of 51.3% were married (N = 104,171). Over half of the patients were white (76.7%, N = 155,877). Most patients were insured (83.0%, N = 168,577). Regarding the site of the primary tumour, 40.3% (N = 81,903) of cancers were located in the right colon, 33.3% (N = 67,565) were in the left colon, and 23.5% (N = 47,660) were in the rectum. Most CRC patients were diagnosed at grade III (59.1%, N = 111,971), N0 (58.5%, N = 118,929) and stage T3 (41.7%, N = 84,747). The detailed demographic and clinical characteristics are displayed in Table 1.

**Table 1:** Logistic regression for characteristics to develop initial lung metastasis in patients with colorectal cancer (diagnosed 2010–2016)

Subject characteristics	Patients’ No. of CRC (2010–2016) (N = 203,138)			Univariable analysis		Multivariable analysis^a^	
	LM	Entire cohort	%	OR [95% CI]	*P* value	OR [95% CI]	*P* value
*Age(years)*							
≤ 50	1587	31,717	5.0	1 (Reference)	1.00	1 (Reference)	1.00
51–60	2517	46,551	5.4	1.09 (1.02–1.16)	0.013	1.11 (1.03–1.19)	0.007
61–70	2828	51,891	5.6	1.09 (1.03–1.17)	0.005	1.24 (1.16–1.34)	< 0.001
71–80	2088	40,878	5.1	1.02 (0.96–1.09)	0.525	1.34 (1.24–1.45)	< 0.001
81–90	1319	27,329	4.8	0.96 (0.89–1.04)	0.321	1.24 (1.14–1.36)	< 0.001
≥ 91	259	4772	5.4	1.09 (0.95–1.25)	0.213	1.13 (0.96–1.32)	0.132
*Sex*							
Female	4855	97,411	5.0	1 (Reference)	1.00	1 (Reference)	1.00
Male	5743	105,727	5.4	1.10 (1.05–1.14)	< 0.001	0.93 (0.84–1.03)	0.142
*Race*							
White	7795	155,877	5.0	1 (Reference)	1.00	1 (Reference)	1.00
Black	1702	25,423	6.7	1.36 (1.29–1.44)	< 0.001	1.12 (1.05–1.19)	0.001
Others^b^	1078	19,755	5.5	1.10 (1.03–1.17)	0.006	1.09 (1.01–1.18)	0.022
Unknown	23	2083	1.1	0.21 (0.14–0.32)	< 0.001	0.33 (0.21–0.50)	< 0.001
*Marital status*							
Unmarried^c^	5136	85,861	6.0	1 (Reference)	1.00	1 (Reference)	1.00
Married	4884	104,171	4.7	0.77 (0.74–0.80)	< 0.001	0.91 (0.87–0.95)	< 0.001
Unknown	578	13,106	4.4	0.73 (0.66–0.79)	< 0.001	0.93 (0.84–1.03)	0.139
*Insurance status*							
Insured	8087	168,577	4.8	1 (Reference)	1.00	1 (Reference)	1.00
Uninsured	589	6818	8.6	1.48 (1.40–1.56)	< 0.001	1.11 (1.05–1.18)	0.001
Any Medic aid	1922	27,743	6.9	1.88 (1.72–2.05)	< 0.001	1.35 (1.22–1.50)	< 0.001
*Site*							
Right colon	3107	81,903	3.8	1 (Reference)	1.00	1 (Reference)	1.00
Left colon	3568	67,565	5.3	1.41 (1.35–1.49)	< 0.001	1.23 (1.21–1.35)	< 0.001
Rectum	2898	47,660	6.1	1.64 (1.56–1.73)	< 0.001	1.96 (1.84–2.08)	< 0.001
Unknown	1025	6010	17.1	5.22 (4.83–5.63)	< 0.001	1.32 (1.20–1.45)	< 0.001
*Histological grade*							
Grade I	440	20,942	2.1	1 (Reference)	1.00	1 (Reference)	1.00
Grade II	4796	119,971	4.0	1.94 (1.76–2.14)	< 0.001	1.35 (1.21–1.50)	< 0.001
Grade III	1377	26,680	5.2	2.54 (2.27–2.83)	< 0.001	1.25 (1.10–1.40)	< 0.001
Grade IV	224	5430	4.1	2.01 (1.70–2.36)	< 0.001	1.12 (0.93–1.34)	0.229
Unknown	3761	30,115	12.5	6.65 (6.01–7.35)	< 0.001	1.87 (1.67–2.09)	< 0.001
*Lymphatic metastasis*							
N0	3631	118,929	3.1	1 (Reference)	1.00	1 (Reference)	1.00
N1	3435	49,501	6.9	2.37 (2.26–2.48)	< 0.001	1.74 (1.64–1.85)	< 0.001
N2	1609	25,083	6.4	2.18 (2.05–2.31)	< 0.001	1.61 (1.49–1.74)	< 0.001
Unknown	1923	9625	20.0	7.93 (7.47–8.42)	< 0.001	1.58 (1.47–1.70)	< 0.001
*T stage*							
T1	1121	37,387	3.0	1 (Reference)	1.00	1 (Reference)	1.00
T2	175	22,697	0.8	0.25 (0.21–0.30)	< 0.001	0.36 (0.30–0.42)	< 0.001
T3	2627	84,747	3.1	1.04 (0.96–1.11)	0.343	0.74 (0.68–0.80)	< 0.001
T4	2218	30,766	7.2	2.51 (2.34–2.71)	< 0.001	1.16 (1.07–1.27)	0.001
Unknown	4457	27,541	16.2	6.25 (5.84–6.68)	< 0.001	1.80 (1.66–1.94)	< 0.001
*CEA*							
Negative	899	57,688	1.6	1 (Reference)	1.00	1 (Reference)	1.00
Positive	6325	53,812	11.8	8.41 (7.84–9.03)	< 0.001	2.40 (2.22–2.59)	< 0.001
Unknown	3374	91,638	3.7	2.42 (2.24–2.60)	< 0.001	1.39 (1.28–1.51)	< 0.001
*Liver metastasis*							
No	2863	172,420	1.7	1 (Reference)	1.00	1 (Reference)	1.00
Yes	7608	30,258	25.1	19.89 (19.02–20.81)	< 0.001	9.13 (8.66–9.62)	< 0.001
Unknown	127	460	27.6	22.59 (18.35–27.8)	< 0.001	4.95 (3.83–6.40)	< 0.001
*Bone metastasis*							
No	9158	199,953	4.6	1 (Reference)	1.00	1 (Reference)	1.00
Yes	1089	2454	44.4	16.62 (15.31–18.05)	< 0.001	3.53 (3.21–3.88)	< 0.001
Unknown	351	731	48.0	19.24 (16.62–22.28)	< 0.001	2.32 (1.80–2.99)	< 0.001
*Brain metastasis*							
No	9899	201,765	4.9	1 (Reference)	1.00	1 (Reference)	1.00
Yes	299	566	52.8	21.71 (18.38–25.63)	< 0.001	8.53 (6.94–10.48)	< 0.001
Unknown	400	807	49.6	19.05 (16.57–21.90)	< 0.001	2.45 (1.94–3.10)	< 0.001

CEA, carcinoembryonic antigen; CRC, colorectal cancer; CI, confidence interval; LM, lung metastasis; NA, not available; OR, odds ratios

^a^Adjusted for age, sex, race, marital status, insurance status, site, histological grade, lymphatic metastasis, T stage, CEA, liver metastasis, bone metastasis, and brain metastasis

^b^Includes American Indian/Alaska Native and Asian or Pacific Islander

^c^Includes single, separated, widowed, and divorced

### Risk factors for developing lung metastasis

The univariate logistic regression analysis showed that age, sex, race, marital status, insurance status, primary site, histological grade, lymphatic metastasis, T stage, CEA, liver metastasis, bone metastasis and brain metastasis were all correlated with the occurrence of lung metastasis. The multivariate logistic regression confirmed that older age, black race, unmarried status, uninsured status, site, poor histological differentiation, more lymphatic metastasis, T4/T1 stage, CEA positivity and liver metastasis, bone metastasis and brain metastasis were associated with lung metastasis (see Table 1). Only sex was not significantly associated with lung metastasis. After excluding stage T1 and stage T2 CRC patients, there were only 143,054 patients remaining. Univariate and multivariate logistic regression analyses were then performed, which revealed that most of the factors were still risk factors for metastasis, and only sex was not significantly associated with lung metastasis. The results are shown in the Additional file 1: Table S1. These data are consistent with the results obtained when stage T1 and stage T2 CRC patients were not excluded (see Table 1).

### Survival estimation and prognostic factors for lung metastasis

**Fig. 2:**
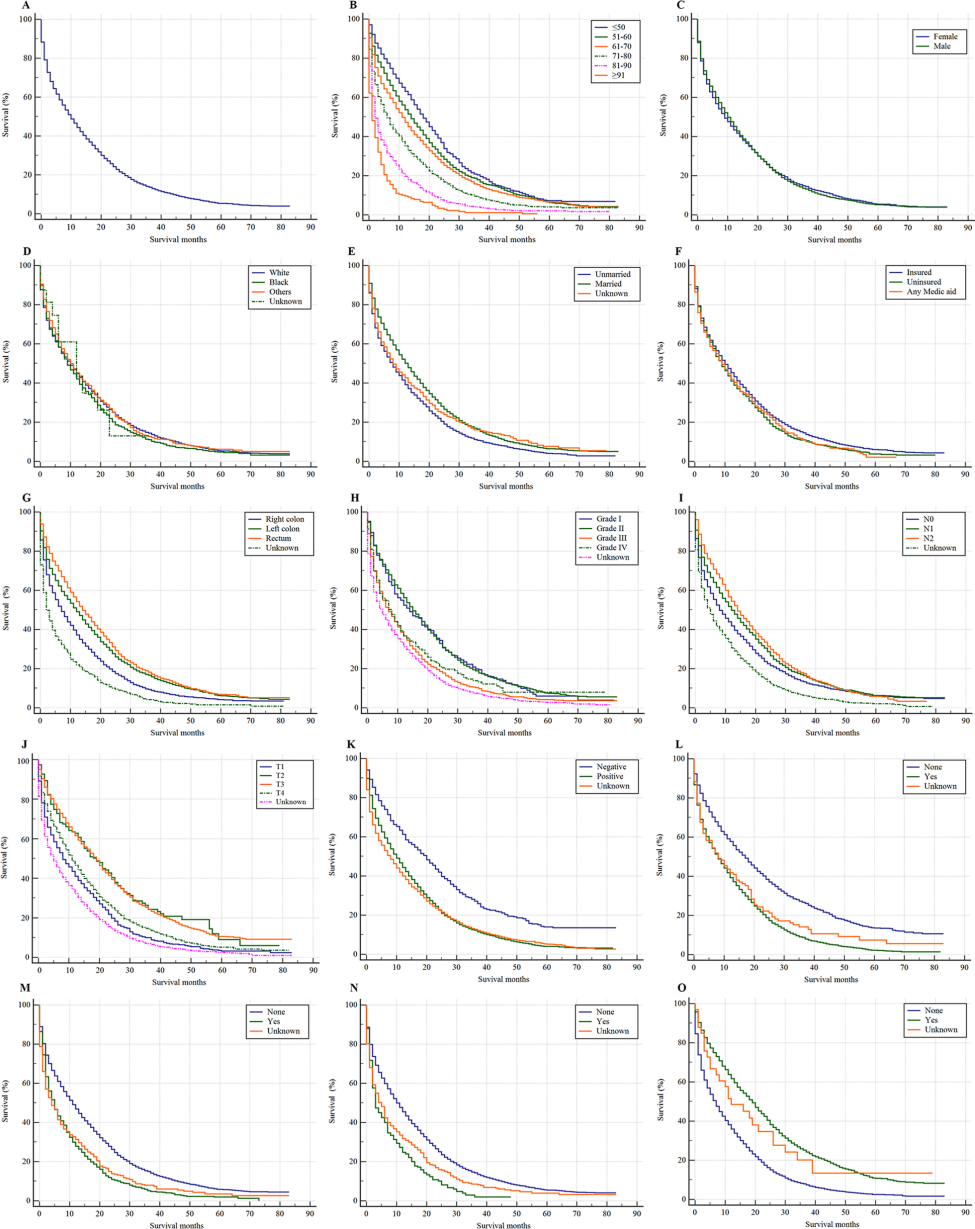
Kaplan–Meier analysis of overall survival for colorectal cancer patients with lung metastasis. Overall (**A**), age (**B**), sex (**C**), race (**D**), marital status (**E**), insurance status (**F**), primary site (**G**), grade (**H**), lymphatic metastasis (**I**), T stage (**J**), CEA (**K**), liver metastasis (**L**), bone metastasis (**M**), brain metastasis (**N**), and surgical treatments of the primary site (**O**)

A total of 8,867 CRC patients diagnosed with lung metastasis between 2010 and 2015 were included to estimate survival and identify prognostic factors. The median survival of CRC patients with lung metastasis was 10.0 months (95% CI 9.6–10.5 months). The 1-year, 3-year, and 5-year survival rates for lung metastasis patients were 44.3%, 13.5%, and 5.2%, respectively. When the cancers were located in the right colon or had poorly differentiated grade, were CEA positive, or involved different metastatic organs, the median survival of lung metastasis patients was reduced (see Table 2). CRC patients with lung metastasis who received surgery had longer median survival times than those who did not have surgery (19.0 months vs. 7.0 months, *P* < 0.001). Kaplan–Meier analysis was performed for CRC patients with lung metastasis (Fig. 2A, overall). The overall survival outcomes of patients stratified by age (Fig. 2B), sex (Fig. 2C), race (Fig. 2D), marital status (Fig. 2E), insurance status (Fig. 2F), primary site (Fig. 2G), grade (Fig. 2H), lymphatic metastasis (Fig. 2I), T stage (Fig. 2J), CEA (Fig. 2K), liver metastasis (Fig. 2L), bone metastasis (Fig. 2M), brain metastasis (Fig. 2N), and surgical treatments of the primary site (Fig. 2O) are shown in Fig. 2.

**Table 2:** Cox regression for analyzing the mortality among lung metastasis patients in colorectal cancer (diagnosed 2010–2015)

Subject characteristics	No. of CRC patients with LM (N = 8867)		Survival, median (95% CI, month)	Univariable analysis		Multivariable analysis^a^	
	Overall	Deceased (rate, %)		HR [95% CI]	*P* value	HR [95% CI]	*P*-value
*Age(years)*							
≤ 50	1313	1007 (76.7)	18 (16.7–19.3)	1 (Reference)	1.00	1 (Reference)	1.00
51–60	2115	1698 (80.3)	14 (13.0–15.0)	1.18 (1.09–1.27)	< 0.001	1.14 (1.05–1.23)	0.001
61–70	2353	1937 (82.3)	11 (10.2–11.9)	1.32 (1.22–1.42)	< 0.001	1.31 (1.21–1.41)	< 0.001
71–80	1745	1549 (88.8)	6 (5.3–6.8)	1.76 (1.62–1.90)	< 0.001	1.82 (1.68–1.98)	< 0.001
81–90	1121	1062 (94.7)	2 (1.7–2.3)	2.69 (2.46–2.93)	< 0.001	2.72 (2.48–2.98)	< 0.001
≥ 91	220	216 (98.2)	1 (0.4–1.6)	3.89 (3.36–4.51)	< 0.001	3.61 (3.09–4.21)	< 0.001
*Sex*							
Female	4068	3416 (84.0)	9 (8.3–9.7)	1 (Reference)	1.00	-	-
Male	4799	4053 (84.5)	10 (9.4–10.6)	0.99 (0.95–1.04)	0.787	-	-
*Race*							
White	6525	5475 (83.9)	10 (9.5–10.6)	1 (Reference)	1.00	1 (Reference)	1.00
Black	1424	1233 (86.6)	9 (8.0–10.0)	1.08 (1.01–1.15)	0.016	1.06 (1.00–1.13)	0.060
Others^b^	902	750 (83.2)	10 (8.6–11.4)	0.98 (0.90–1.05)	0.542	1.02 (0.94–1.10)	0.690
Unknown	16	11 (68.8)	12 (2.7–21.3)	0.96 (0.53–1.73)	0.885	1.14 (0.63–2.07)	0.663
*Marital status*							
Unmarried^c^	4315	3739 (86.7)	8 (7.4–8.6)	1 (Reference)	1.00	1 (Reference)	1.00
Married	4060	3320 (81.8)	13 (12.3–13.7)	0.79 (0.75–0.82)	< 0.001	0.87 (0.83–0.91)	< 0.001
Unknown	492	410 (83.3)	8 (6.0–10.0)	0.87 (0.78–0.96)	0.005	0.92 (0.83–1.02)	0.119
*Insurance status*							
Insured	6763	5665 (83.8)	10 (9.5–10.5)	1 (Reference)	1.00	1 (Reference)	1.00
Uninsured	1598	1376 (86.1)	9 (8.1–9.9)	1.10 (1.00–1.21)	0.058	1.24 (1.16–1.32)	< 0.001
Any Medic aid	506	428 (84.6)	9 (7.1–10.9)	1.11 (1.05–1.18)	0.001	1.35 (1.22–1.49)	< 0.001
*Site*							
Right colon	2617	2309 (88.2)	7 (6.3–7.7)	1 (Reference)	1.00	1 (Reference)	1.00
Left colon	2986	2435 (81.6)	12 (11.1–12.9)	0.76 (0.72–0.81)	< 0.001	0.82 (0.77–0.87)	< 0.001
Rectum	2399	1922 (80.1)	14 (13.1–15.0)	0.69 (0.64–0.73)	< 0.001	0.68 (0.64–0.73)	< 0.001
Unknown	865	803 (92.8)	2 (1.5–2.5)	1.51 (1.39–1.63)	< 0.001	1.11 (1.02–1.21)	0.018
*Histological grade*							
Grade I	356	280 (78.7)	14 (11.1–16.9)	1 (Reference)	1.00	1 (Reference)	1.00
Grade II	4050	3202 (79.1)	15 (14.2–15.8)	0.99 (0.87–1.11)	0.810	1.08 (0.95–1.22)	0.244
Grade III	1167	1031 (88.4)	7 (6.1–7.9)	1.49 (1.31–1.71)	< 0.001	1.58 (1.38–1.81)	< 0.001
Grade IV	195	166 (85.1)	8 (5.6–10.4)	1.34 (1.11–1.63)	0.003	1.51 (1.24–1.83)	< 0.001
Unknown	3099	2790 (90.0)	5 (4.5–5.5)	1.78 (1.57–2.01)	< 0.001	1.32 (1.16–1.49)	< 0.001
*Lymphatic metastasis*							
N0	3021	2542 (84.1)	8 (7.2–8.8)	1 (Reference)	1.00	1 (Reference)	1.00
N1	2938	2388 (81.3)	12 (11.1–12.9)	0.85 (0.81–0.90)	< 0.001	1.02 (0.96–1.08)	0.485
N2	1315	1078 (82.0)	14 (12.7–15.3)	0.79 (0.74–0.85)	< 0.001	1.17 (1.07–1.27)	< 0.001
Unknown	1593	1461 (91.7)	8 (7.2–8.8)	1.37 (1.28–1.46)	< 0.001	1.07 (1.00–1.14)	0.068
*T stage*							
T1	1052	920 (87.5)	8 (6.7–9.3)	1 (Reference)	1.00	1 (Reference)	1.00
T2	151	114 (75.5)	19 (14.2–23.8)	0.48 (0.37–0.63)	< 0.001	0.78 (0.60–1.02)	0.071
T3	2285	1717 (75.1)	19 (17.9–20.1)	0.75 (0.69–0.82)	< 0.001	0.93 (0.85–1.02)	0.118
T4	1875	1572 (83.8)	11 (10.1–11.9)	0.83 (0.75–0.92)	< 0.001	1.07 (0.96–1.18)	0.223
Unknown	3504	3146 (89.8)	5 (4.5–5.5)	1.23 (1.13–1.35)	< 0.001	1.01 (0.92–1.11)	0.792
*CEA*							
Negative	745	543 (72.9)	20 (17.7–22.3)	1 (Reference)	1.00	1 (Reference)	1.00
Positive	5282	4477 (84.8)	10 (9.5–10.6)	1.62 (1.48–1.77)	< 0.001	1.27 (1.16–1.39)	< 0.001
Unknown	2840	2449 (86.2)	8 (7.2–8.8)	1.73 (1.57–1.89)	< 0.001	1.34 (1.21–1.47)	< 0.001
*Liver metastasis*							
No	2406	1759 (73.1)	17 (15.8–18.2)	1 (Reference)	1.00	1 (Reference)	1.00
Yes	6350	5612 (88.4)	8 (7.5–8.5)	1.75 (1.66–1.85)	< 0.001	1.64 (1.55–1.73)	< 0.001
Unknown	111	98 (88.3)	8 (3.5–12.5)	1.52 (1.24–1.86)	< 0.001	1.21 (0.97–1.50)	0.098
*Bone metastasis*							
No	7663	6357 (83.0)	11 (10.5–11.5)	1 (Reference)	1.00	1 (Reference)	1.00
Yes	899	829 (92.2)	5 (4.2–5.8)	1.55 (1.45–1.67)	< 0.001	1.38 (1.28–1.48)	< 0.001
Unknown	305	283 (92.8)	4 (2.6–5.4)	1.47 (1.30–1.65)	< 0.001	1.32 (1.09–1.60)	0.004
*Brain metastasis*							
No	8267	6921 (83.7)	10 (9.5–10.5)	1 (Reference)	1.00	1 (Reference)	1.00
Yes	248	227 (91.5)	3 (2.0–4.0)	1.68 (1.47–1.92)	< 0.001	1.51 (1.32–1.73)	< 0.001
Unknown	352	321 (91.2)	5 (3.8–6.3)	1.36 (1.22–1.52)	< 0.001	0.86 (0.72–1.03)	0.103
*Surg (pri)*							
No	5956	5286 (88.8)	7 (6.6–7.4)	1 (Reference)	1.00	1 (Reference)	1.00
Yes	2878	2157 (75.0)	19 (17.9–20.1)	0.51 (0.49–0.54)	< 0.001	0.56 (0.52–0.60)	< 0.001
Unknown	33	26 (78.8)	12 (3.5–20.5)	0.59 (0.40–0.87)	0.007	0.45 (0.31–0.67)	< 0.001

CEA, carcinoembryonic antigen; CRC, colorectal cancer; CI, confidence interval; HR, hazard ratio; LM, lung metastasis; NA, not available; Surg(pri), surgical treatments of primary site

^a^Adjusted for age, race, marital status, insurance status, site, histological grade, lymphatic metastasis, T stage, CEA, liver metastasis, bone metastasis, and brain metastasis

^b^Includes American Indian/Alaska Native and Asian or Pacific Islander

^c^Includes single, separated, widowed, and divorced

The univariate analysis suggested that older age, unmarried status, insurance status, right colon, poor histological differentiation, N stage, T stage, CEA positivity, liver metastasis, bone metastasis, brain metastasis and no surgical treatments of the primary tumour were associated with poor prognosis. Multivariable Cox regression confirmed that older age, unmarried status, uninsured status, right colon, poor histological differentiation, more lymphatic metastasis, positive CEA, liver metastasis, bone metastasis, brain metastasis and no surgical treatments of the primary tumour were all risk factors for poorer prognosis. See Table 2 for more details.

### Homogeneous and heterogeneous risk and prognostic factors

**Fig. 3:**
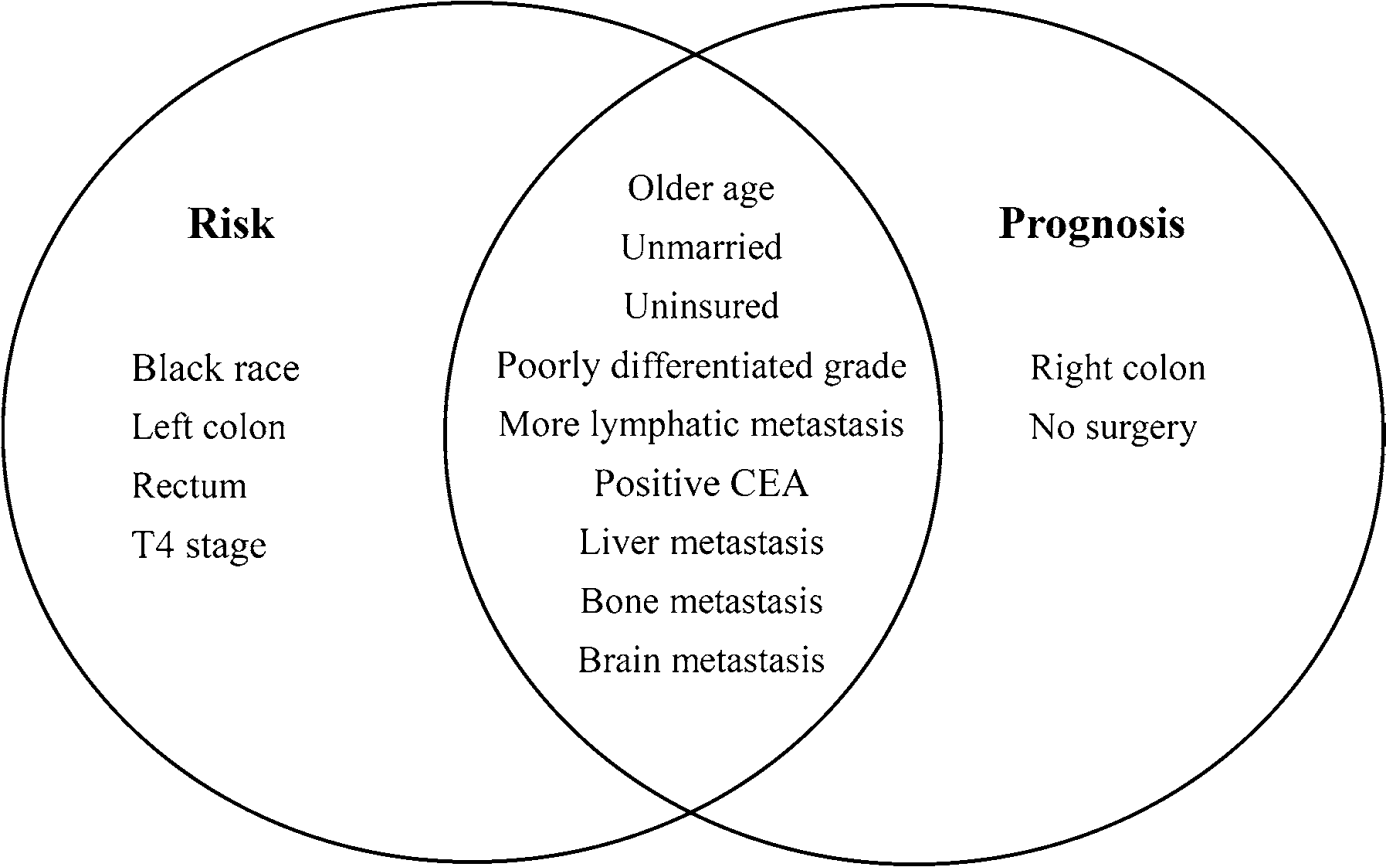
Homogeneous and heterogeneous risk factors for the occurrence and prognosis of synchronous lung metastasis in colorectal cancer

According to the results of multivariable logistic regression and multivariable Cox regression analyses, the homogeneous lung metastasis risk and prognostic factors in CRC were older age, unmarried status, uninsured status, poor histological differentiation, more lymphatic metastasis, CEA positivity, liver metastasis, bone metastasis, and brain metastasis. However, patients with black race, left colon, rectum, and T4 stage disease were more likely to develop lung metastasis, while patients with right colon disease without surgical treatment of primary tumours had poor survival outcomes (Fig. 3).

## Discussion

We investigated the incidence of synchronous lung metastasis in newly diagnosed CRC patients using SEER database information. Synchronous lung metastasis occurred in 5.2% of CRC patients. This incidence was lower than those reported in Mitry’s study (11.3%) [5] and Yahagi’s study (6.9%) [10] but higher than those reported in Huang’s study (2.4%) [6]. This is presumably due to the different sample sizes of the study population. In addition, the SEER database only records confirmed patients who have been comprehensively evaluated, and some asymptomatic patients may be missed. Therefore, the incidence in this study may be underestimated.

Accurately identifying the population at high risk for lung metastasis is helpful for subsequent individualized treatment. Our results showed that patients with older age, black race, left colon, rectum, poorly differentiated grade, more lymphatic metastasis, T4 stage, CEA positivity and liver metastasis, bone metastasis and brain metastasis were more likely to develop lung metastasis. Given that stage T1 and stage T2 CRC patients have much lower risk of metastasis, another univariate and multivariate logistic regression analyses were performed which only included patients with stage T3 and stage T4 CRC (see Additional file 1: Table S1). We found the results were comparable when the sample was restricted to stages T3 and stage T4 patients. This also suggests that it is necessary to screen lung metastasis in CRC patients with higher T stage. In addition, unmarried and uninsured patients were also at high risk for lung metastasis. Nevertheless, previous studies did not investigate the impact of marital status and insurance status on the incidence of lung metastasis [5, 6]. A 30-year population-based study found that only the primary site of CRC was significantly associated with synchronous lung metastasis [5]. The results of another study are similar to ours, except for marital status and insurance status [6]. Therefore, the relationship between marital status, insurance status, and lung metastasis incidence warrants further investigation. Regardless, patients with the above risk factors are recommended for lung metastasis screening.

In addition to risk factors, identifying prognostic factors is important in cancer management. We found 11 prognostic factors, including older age, unmarried status, insurance status, right colon, poor histological differentiation, more lymphatic metastasis, CEA positivity, liver metastasis, bone metastasis, brain metastasis and no surgery. Surprisingly, there was no significant correlation between T stage and prognosis of lung metastasis patients, which was consistent with the findings of Huang’s study [6]. Notably, the survival time in patients with stage T1 disease was lower than that in patients with stage T2 to T4 disease. In addition, T stage was found to be a prognostic factor in the univariate Cox regression, while it became a nonsignificant factor in the multivariate Cox regression. T stage has also been found not to be a prognostic factor in patients with brain metastasis [11]. Therefore, we conclude that T stage cannot be used to estimate survival in CRC patients. However, T stage has been reported as an independent prognostic factor in CRC patients with liver metastasis or bone metastasis [9, 12]. Accordingly, the relationship between T stage and the prognosis of distant metastases in CRC patients is still controversial and requires further investigation.

Based on the analysis of risk and prognostic factors, the most important findings in this research were the nine homogeneous factors. To the best of our knowledge, this is the first report to describe the homogeneous factors associated with lung metastasis in CRC patients. These factors can be used to predict the occurrence of lung metastasis, estimate the prognosis, and improve lung metastasis screening for CRC patients. Among the nine homogeneous factors, different metastatic organs ranked much higher in both odds ratio and hazard ratio. Liver metastasis ranked highest, followed by bone metastasis and brain metastasis, indicating that lung metastasis was closely related to liver metastasis. One previous study found that the expression of several key genes plays an important role in determining the distant metastasis of CRC to these two organs [13, 14]. However, the specific molecular mechanisms by which CRC cells affect the liver and lung remain unclear and need to be further studied [15]. Nonetheless, our results suggest that routine liver scanning is necessary for patients with lung metastatic CRC.

In terms of heterogeneous factors, we found that patients with tumours located in the left colon and rectum were more likely to develop lung metastasis, which was consistent with the results of Qiu’s study [3]. The results of this study also showed that patients with lung metastasis from right colon cancer had worse survival than those with metastasis from left colon cancer. This finding is also consistent with the results of previous studies [16–18]. In addition to the primary site, surgical treatment is another heterogeneous factor. Patients who underwent surgical resection of the primary tumour survived longer than those who did not [19]. Some studies have shown that resection of lung metastasis also has a positive effect on improving survival outcomes [20–23]. Therefore, surgical resection of both the primary tumour and the metastasis is an effective measure for CRC patients with lung metastasis.

This study has some limitations. Only patients with synchronous lung metastasis were studied, and the incidence and prognosis of patients with metachronous lung metastasis are still unclear. Meanwhile, the incidence of lung metastasis may be underestimated. In addition, since other important information, including chemotherapy and radiotherapy, was not available from the SEER database, their impact on survival in CRC patients still needs to be further studied. Despite these limitations, our study based on a large cohort of CRC patients demonstrated homogeneous and heterogeneous risk and prognostic factors for lung metastasis. These findings may be helpful for clinicians to identify high-risk patients and improve lung metastasis screening for CRC patients.

## Conclusion

In this study, we found that the incidence of lung metastasis in CRC patients was 5.2%, and the median survival of CRC patients with lung metastasis was 10.0 months. Some lung metastasis risk and prognostic factors were found. A total of nine homogeneous risk factors and several heterogeneous factors were identified. These results are helpful for clinicians to conduct clinical evaluations and individualize treatment strategies.

## Supplementary Information

**Additional file 1**. **Table S1.** Logistic regression for characteristics to develop initial lung metastasis in patients with colorectal cancer (excluding stage T1 and stage T2 patients).

## Data Availability

The data that support the fndings of this study are available from the corresponding author upon reasonable request.

## Abbreviations

CEA: Carcinoembryonic antigen
CRC: Colorectal cancer
NCI: National Cancer Institute
SEER: Surveillance, Epidemiology, and End Results
SD: Standard deviation
SPSS: Statistical Package for the Social Sciences
