# Correction: Homogeneous and heterogeneous risk and prognostic factors for lung metastasis in colorectal cancer patients

**DOI:** 10.1186/s12876-022-02306-w

**Published:** 2022-05-17

**Authors:** Hongmei Wang, Xuefeng Shan, Min Zhang, Kun Qian, Zhengze Shen, Weiying Zhou

**Affiliations:** 1 Department of Pharmacology, College of Pharmacy, Chongqing Medical University, 1 Yixueyuan Road, Yuzhong District, Chongqing, 400016 China; 2 Chongqing Key Laboratory of Drug Metabolism, Chongqing Medical University, Chongqing, 400016 China; 3 Key Laboratory for Biochemistry and Molecular Pharmacology of Chongqing, Chongqing Medical University, Chongqing, 400016 China; 4 Department of Pharmacy, The First Affiliated Hospital of Chongqing Medical University, Chongqing, 400016 China; 5 Department of Epidemiology and Health Statistics, School of Public Health and Management, Chongqing Medical University, Chongqing, 400016 China; 6 Department of Gastrointestinal Surgery, The First Affiliated Hospital of Chongqing Medical University, Chongqing, 400016 China; 7 Department of Pharmacy, Yongchuan Hospital of Chongqing Medical University, 439 Xuanhua Road, Yongchuan District, Chongqing, 402160 China

## Correction to: BMC Gastroenterology (2022) 22:193 10.1186/s12876-022-02270-5

After publication of this article [1], the authors reported that in this article there is a superfluous line in Fig. 1; the figure should have appeared as shown below.

**Fig. 1:**
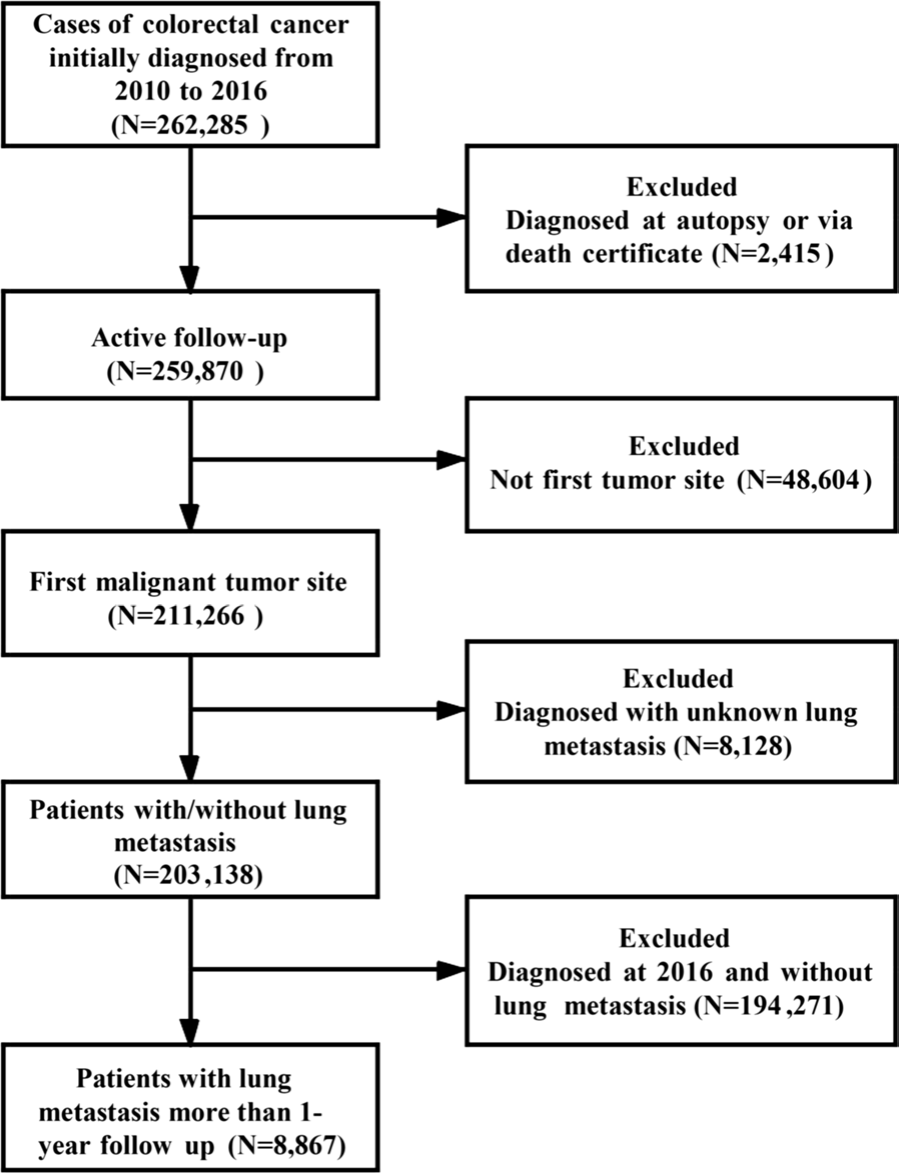
Flowchart of colorectal cancer patient selection

The original article [1] has been updated.
