# Supplementary material for: Homogeneous and heterogeneous risk and prognostic factors for lung metastasis in colorectal cancer patients
Source: BMC Gastroenterol. 2022 Apr 18;22:193. doi: 10.1186/s12876-022-02270-5 (PMC9016976; doi:10.1186/s12876-022-02270-5)
Supplement: Supplementary file 1 — Additional file 1. Table S1. Logistic regression for characteristics to develop initial lung metastasis in patients with colorectal cancer (excluding stage T1 and stage T2 patients). [file 12876_2022_2270_MOESM1_ESM.docx]

**SUPPORTING INFORMATION**

Table S1. Logistic regression for characteristics to develop initial lung metastasis in patients with colorectal cancer (excluding stage T1 and stage T2 patients)

| **Subject characteristics** | **Patients’ No. of CRC (2010-2016)**  **(N = 143,054)** | | | **Univariable** | | | **Multivariable^a^** | | |  |
| --- | --- | --- | --- | --- | --- | --- | --- | --- | --- | --- |
|  | **LM** | **Entire cohort** | **%** | | **OR [95% CI]** | **P value** | | **OR [95% CI]** | **P value** | |
| **Age(years)** |  |  |  | |  |  | |  |  | |
| ≤50 | 1393 | 22120 | 6.3 | | 1(Reference) | 1.00 | | 1(Reference) | 1.00 | |
| 51-60 | 2205 | 31720 | 7.0 | | 1.11(1.04-1.19) | 0.003 | | 1.10(1.02-1.19) | 0.018 | |
| 61-70 | 2484 | 35972 | 6.9 | | 1.10(1.03-1.18) | 0.004 | | 1.23(1.14-1.33) | ﹤0.001 | |
| 71-80 | 1834 | 28947 | 6.3 | | 1.01(0.94-1.08) | 0.860 | | 1.31(1.20-1.42) | ﹤0.001 | |
| 81-90 | 1158 | 20539 | 5.6 | | 0.89(0.82-0.96) | 0.004 | | 1.16(1.06-1.27) | 0.002 | |
| ≥91 | 228 | 3756 | 6.1 | | 0.96(0.83-1.11) | 0.595 | | 1.02(0.87-1.21) | 0.803 | |
| **Sex** |  |  |  | |  |  | |  |  | |
| Female | 4280 | 68438 | 6.3 | | 1(Reference) | 1.00 | | 1(Reference) | 1.00 | |
| Male | 5022 | 74616 | 6.7 | | 1.08(1.04-1.13) | ﹤0.001 | | 0.96(0.91-1.01) | 0.079 | |
| **Race** |  |  |  | |  |  | |  |  | |
| White | 6858 | 110056 | 6.2 | | 1(Reference) | 1.00 | | 1(Reference) | 1.00 | |
| Black | 1480 | 17963 | 8.2 | | 1.35(1.27-1.43) | ﹤0.001 | | 1.10(1.03-1.17) | 0.007 | |
| Others^b^ | 945 | 13839 | 6.8 | | 1.10(1.03-1.18) | 0.006 | | 1.09(1.00-1.18) | 0.043 | |
| Unknown | 19 | 1196 | 1.6 | | 0.24(0.15-0.38) | ﹤0.001 | | 0.30(0.19-0.49) | ﹤0.001 | |
| **Marital status** |  |  |  | |  |  | |  |  | |
| Unmarried^c^ | 4505 | 62840 | 7.2 | | 1(Reference) | 1.00 | | 1(Reference) | 1.00 | |
| Married | 4275 | 71738 | 6.0 | | 0.82(0.79-0.86) | ﹤0.001 | | 0.92(0.87-0.97) | 0.001 | |
| Unknown | 522 | 8476 | 6.2 | | 0.85(0.77-0.93) | 0.001 | | 0.97(0.87-1.08) | 0.584 | |
| **Insurance status** |  |  |  | |  |  | |  |  | |
| Insured | 7098 | 116827 | 6.1 | | 1(Reference) | 1.00 | | 1(Reference) | 1.00 | |
| Uninsured | 1681 | 20792 | 8.1 | | 1.36(1.29-1.44) | ﹤0.001 | | 1.09(1.02-1.16) | 0.008 | |
| Any Medic aid | 523 | 5435 | 9.6 | | 1.65(1.50-1.81) | ﹤0.001 | | 1.31(1.18-1.46) | ﹤0.001 | |
| **Site** |  |  |  | |  |  | |  |  | |
| Right colon | 2734 | 59819 | 4.6 | | 1(Reference) | 1.00 | | 1(Reference) | 1.00 | |
| Left colon | 3144 | 47198 | 6.7 | | 1.49(1.41-1.57) | ﹤0.001 | | 1.30(1.23-1.38) | ﹤0.001 | |
| Rectum | 2441 | 30626 | 8.0 | | 1.81(1.71-1.91) | ﹤0.001 | | 1.97(1.84-2.10) | ﹤0.001 | |
| Unknown | 983 | 5411 | 18.2 | | 4.64(4.28-5.02) | ﹤0.001 | | 1.34(1.22-1.47) | ﹤0.001 | |
| **Histological grade** |  |  |  | |  |  | |  |  | |
| Grade I | 380 | 9777 | 3.9 | | 1(Reference) | 1.00 | | 1(Reference) | 1.00 | |
| Grade II | 4161 | 84105 | 4.9 | | 1.29(1.16-1.43) | ﹤0.001 | | 1.13(1.00-1.27) | 0.043 | |
| Grade III | 1225 | 22473 | 5.5 | | 1.43(1.27-1.60) | ﹤0.001 | | 1.01(0.89-1.15) | 0.901 | |
| Grade IV | 212 | 4746 | 4.5 | | 1.16(0.97-1.37) | 0.097 | | 0.94(0.78-1.13) | 0.504 | |
| Unknown | 3324 | 21953 | 15.1 | | 4.41(3.96-4.92) | ﹤0.001 | | 1.55(1.37-1.75) | ﹤0.001 | |
| **Lymphatic metastasis** |  |  |  | |  |  | |  |  | |
| N0 | 2891 | 69011 | 4.2 | | 1(Reference) | 1.00 | | 1(Reference) | 1.00 | |
| N1 | 3029 | 41966 | 7.2 | | 1.78(1.69-1.88) | ﹤0.001 | | 1.66(1.56-1.77) | ﹤0.001 | |
| N2 | 1558 | 23878 | 6.5 | | 1.60(1.50-1.70) | ﹤0.001 | | 1.59(1.47-1.72) | ﹤0.001 | |
| Unknown | 1824 | 8199 | 22.2 | | 6.54(6.14-6.98) | ﹤0.001 | | 1.57(1.46-1.70) | ﹤0.001 | |
| **T stage** |  |  |  | |  |  | |  |  | |
| T3 | 2627 | 84747 | 3.1 | | 1(Reference) | 1.00 | | 1(Reference) | 1.00 | |
| T4 | 2218 | 30766 | 7.2 | | 2.43(2.29-2.57) | ﹤0.001 | | 1.62(1.52-1.72) | ﹤0.001 | |
| Unknown | 4457 | 27541 | 16.2 | | 6.04(5.74-6.35) | ﹤0.001 | | 2.50(2.33-2.68) | ﹤0.001 | |
| **CEA** |  |  |  | |  |  | |  |  | |
| Negative | 778 | 38747 | 2.0 | | 1(Reference) | 1.00 | | 1(Reference) | 1.00 | |
| Positive | 5538 | 46271 | 12 | | 6.64(6.15-7.16) | ﹤0.001 | | 2.28(2.09-2.47) | ﹤0.001 | |
| Unknown | 2986 | 58036 | 5.1 | | 2.65(2.44-2.87) | ﹤0.001 | | 1.42(1.31-1.55) | ﹤0.001 | |
| **Liver metastasis** |  |  |  | |  |  | |  |  | |
| No | 2522 | 115852 | 2.2 | | 1(Reference) | 1.00 | | 1(Reference) | 1.00 | |
| Yes | 6666 | 26793 | 24.9 | | 14.88(14.18-15.62) | ﹤0.001 | | 7.82(7.40-8.26) | ﹤0.001 | |
| Unknown | 114 | 409 | 27.9 | | 17.37(13.94-21.63) | ﹤0.001 | | 4.05(3.09-5.32) | ﹤0.001 | |
| **Bone metastasis** |  |  |  | |  |  | |  |  | |
| No | 8029 | 140262 | 5.7 | | 1(Reference) | 1.00 | | 1(Reference) | 1.00 | |
| Yes | 947 | 2134 | 44.4 | | 13.14(12.03-14.35) | ﹤0.001 | | 3.34(3.02-3.69) | ﹤0.001 | |
| Unknown | 326 | 658 | 49.5 | | 16.17(13.86-18.87) | ﹤0.001 | | 2.23(1.71-2.91) | ﹤0.001 | |
| **Brain metastasis** |  |  |  | |  |  | |  |  | |
| No | 8670 | 141839 | 6.1 | | 1(Reference) | 1.00 | | 1(Reference) | 1.00 | |
| Yes | 258 | 490 | 52.7 | | 17.08(14.29-20.42) | ﹤0.001 | | 7.94(6.39-9.88) | ﹤0.001 | |
| Unknown | 374 | 725 | 51.6 | | 16.37(14.13-18.96) | ﹤0.001 | | 2.74(2.14-3.51) | ﹤0.001 | |

Abbreviations: CEA=carcinoembryonic antigen, CRC=colorectal cancer, CI = confidence interval, LM=lung metastasis, NA=not available, OR=odds ratios.

a Adjusted for age, sex, race, marital status, insurance status, site, histological grade, lymphatic metastasis, T stage, CEA, liver metastasis, bone metastasis, and brain metastasis.

b Includes American Indian/Alaska Native and Asian or Pacific Islander.

c Includes single, separated, widowed, and divorced.
